# Relation between pre-existing quality management measures and prevention and containment of COVID-19 outbreaks in 159 nursing homes in Tuscany: a mixed methods study

**DOI:** 10.1136/bmjoq-2023-002560

**Published:** 2024-04-30

**Authors:** Mircha Poldrugovac, Sara Barsanti, Emiliano Pardini, Niek S Klazinga, Dionne S Kringos

**Affiliations:** 1 Department of Public and Occupational Health, Amsterdam UMC Locatie Meibergdreef, Amsterdam, The Netherlands; 2 Quality of Care, Amsterdam Public Health Research Institute, Amsterdam, The Netherlands; 3 Management and Health Laboratory, Institute of Management, Sant'Anna School of Advanced Studies, Pisa, Italy

**Keywords:** Long-Term Care, Nursing homes, Quality measurement

## Abstract

**Background:**

Nursing homes were often the focus of COVID-19 outbreaks. Many factors are known to influence the ability of a nursing home to prevent and contain a COVID-19 outbreak. The role of an organisation’s quality management prior to the pandemic is not yet clear. In the Italian region of Tuscany nursing home performance indicators have been regularly collected since before the pandemic, providing the opportunity to better understand this relationship.

**Objectives:**

To test if there is a difference in the results achieved by nursing homes in Tuscany on 13 quality management indicators, when grouped by severity of COVID-19 outbreaks; and to better understand how these indicators may be related to the ability to control COVID-19 outbreaks, from the perspective of nursing homes.

**Methods:**

We used a mixed methods sequential explanatory design. Based on regional and national databases, 159 nursing homes in Tuscany were divided into four groups by outbreak severity. We tested the significance of the differences between the groups with respect to 13 quality management indicators. The potential relation of these indicators to COVID-19 outbreaks was discussed with 29 managers and other nursing homes’ staff through four group interviews.

**Results:**

The quantitative analysis showed significant differences between the groups of nursing homes for 3 of the 13 indicators. From the perspective of nursing homes, the indicators might not be good at capturing important aspects of the ability to control COVID-19 outbreaks. For example, while staffing availability is seen as essential, the staff-to-bed ratio does not capture the turn-over of staff and temporary absences due to positive COVID-19 testing of staff.

**Conclusions:**

Though currently collected indicators are key for overall performance monitoring and improvement, further refinement of the set of quality management indicators is needed to clarify the relationship with nursing homes’ ability to control COVID-19 outbreaks.

**WHAT IS ALREADY KNOWN ON THIS TOPIC**

- Several factors are known to be associated with COVID-19 outbreaks in nursing homes, including facility size and prevalence in the community. The role of an organisation’s quality management prior to the pandemic is not yet clear.

**WHAT THIS STUDY ADDS**

- Commonly used quality indicators for nursing homes are insufficiently fit for the purpose of monitoring preparedness to control the impact of public health emergencies, such as COVID-19 outbreaks.

**HOW THIS STUDY MIGHT AFFECT RESEARCH, PRACTICE OR POLICY**

- In order to monitor the emergency preparedness and resilience of nursing homes, indicators tailored to these purposes should be sought.

## Introduction

The first big COVID-19 outbreak in Europe was identified in Italy in February 2020.^1^ This was the first European country to tackle the epidemic and faced an extremely difficult situation with high mortality rates in the affected areas.^2^ Our knowledge about the prevention and management of SARS-CoV-2 spread and COVID-19 disease has been improving over time, leading to the adoption of several guidelines and recommendations. In Italy, guidelines were provided by national, regional and municipal authorities and were constantly updated as the body of evidence about the pandemic increased.^3^ The second wave, which in Europe is generally associated with the period between September and December 2020, that is, prior to vaccine availability, was characterised by a lower fatality rate as compared with the first wave but also by a much higher incidence of cases.^4^ The older population was particularly affected, with excess mortality being highest in Europe among persons 75 years old and older and COVID-19 lethality rates also increasing with age. Nursing homes, where older people often with chronic conditions are concentrated in limited space, were often the focus of outbreaks, with deadly consequences for many of its residents,.^4–6^ There were 335 nursing homes in Tuscany in December 2020, with 15 293 beds, 90% of which were dedicated to non-self-sufficient persons. This value represents 14.6 places for 1000 residents 65 years old and older in Tuscany, slightly below the Italian average. The non-self-sufficient persons among 65 years old and older in the general population of Tuscany is about 13%.^7^

Several studies have considered the factors associated with COVID-19 outbreaks or mortality in nursing homes.^8–10^ Consistently larger facilities’ size and higher prevalence of COVID-19 in the community are reported as important factors increasing risk. Staffing plays an important role, however, basic indicators such as staff-to-bed ratios must be complemented by considerations about the use of agency staff, compartmentalising of staff, etc.^11 12^ The role of facilities’ architectural characteristics and of ownership aspects, such as public or private, for-profit or non-for-profit status, require a larger body of evidence.^8–10^

The role of quality management has also been investigated by several studies, with reviews concluding that an association with control of COVID-19 in nursing homes has not yet been convincingly proven.^8 10 13^ However, most studies are focused on the USA and the five-star rating system used within the Nursing Home Compare initiative.^14^ Qualitative studies also looked into initiatives that improved the resilience of nursing homes, but they tend not to explore potential pathways of relationship between quality indicators and COVID-19 outbreaks.^15 16^ To improve our understanding of the potential relationship between quality management and COVID-19 in nursing homes in a European context, it is useful to look at other long-term care systems and explore quantitatively and qualitatively what could be the nature of such an association.

Quality management in nursing homes can include a number of dimensions, including considerations about the quality of life of long-term care residents, their empowerment and independence, respect for their dignity and human rights more broadly as well as improvement or prevention of deterioration of their medical condition.^17^ For example, during COVID-19 nursing homes often halted all external visits to nursing home residents. In Tuscany this measure was partly revoked in June 2020.^18^ In this study we focused on quality measures related to health aspects of care, not claiming superiority of any one dimension over the others, but because of its likely higher relevance to COVID-19 prevention and containment capacity. Furthermore, it was important to consider process and structural measures in addition to outcome measures, to have a more complete picture of quality management in nursing homes.

Our aim was to understand how quality management prepared nursing homes in Tuscany, a region in central Italy, to tackle COVID-19 through the following research questions:

- Is there an association between pre-existing quality management measures of nursing homes in Tuscany and the prevention and spread of COVID-19 within these nursing homes?
- How do nursing homes perceive the relation between these measures and prevention and spread of COVID-19?

## Methods

**Figure 1:**
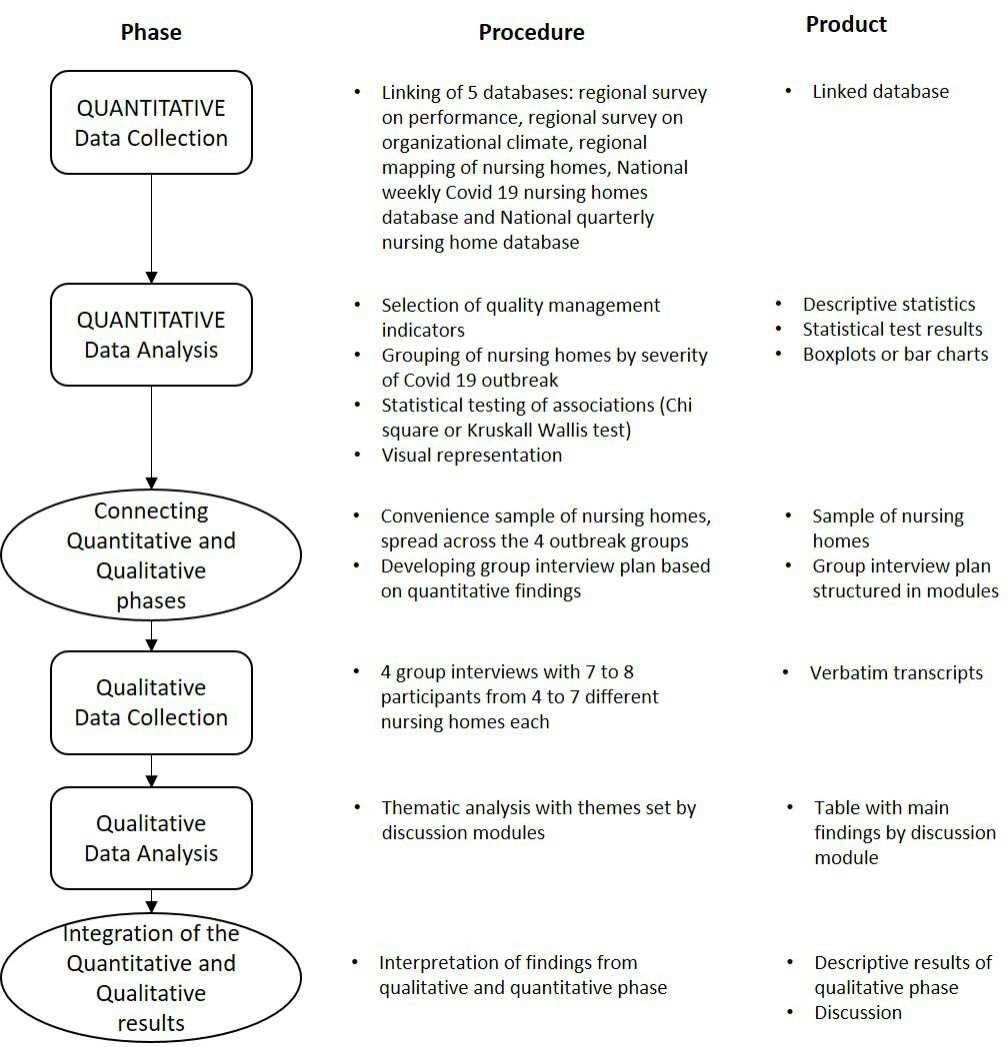
Visual model of the mixed methods study design procedures.

Our study was focused on nursing homes in Tuscany. We investigated the relation between quality management measures related to 2019 and measures for the prevention and containment of COVID-19 outbreaks related to the second half of 2020. We used a mixed methods sequential explanatory design.^19^ This means that the quantitative analysis was undertaken first and the qualitative part of the study followed in order to help explain the quantitative findings (figure 1). The mixed methods approach was chosen for several reasons: COVID-19-related data collection was a novelty for all stakeholders as was the attempt to understand performance data in the context of the pandemic. Therefore, those who provided the data can contribute significantly to understanding what is being measured. Furthermore, the size of the sample and the availability of aggregated data at the facility level limits the possibility for mathematical modelling, hence it is useful to verify with providers of long-term care services the plausibility of any quantitative findings. The research was conducted in line with the requirements for mixed methods research by Lee *et al*.^20^ The related checklist with references is provided in online supplemental file 1.

10.1136/bmjoq-2023-002560.supp1 Supplementary data

### Quantitative phase

#### Data sources

We used several data sources related to nursing homes indicators: (1) structure and process-related nursing home indicators collected biannually by the Management and Healthcare Laboratory (MeS Lab) of the Sant’Anna School for Advanced Studies on behalf of the Tuscany regional authority (Regione Toscana); (2) additional set of data on performance and work climate in nursing homes on a voluntary basis collected annually by the MeS Lab.^21–23^ (3) Data on COVID-19 incidence and prevalence were the weekly data collected by the Italian National Institute for Health (Istituto Superiore di Sanità) between the week starting on 29 June 2020, when data collection began, and the week starting on 28 December 2020, when vaccination of residents began. Since coverage of nursing home was not complete throughout the whole period, only nursing homes that reported data for at least 8 weeks were included. The 8-week time frame was introduced to minimise the likelihood of miscategorising a nursing home as not having an outbreak only because it happened during a period when the nursing home was not reporting data. Additional sources of information for data on personnel and available beds were quarterly reports on nursing homes also provided by the Italian National Institute for Health.^24^ All data sources included facility-level aggregated data. Data has been pulled together matching the data sources to individual nursing homes.

#### Performance indicators

The indicators used in this study were selected from those regularly collected by the MeS Lab. To select indicators three aspects were considered: focusing on healthcare aspects as opposed to other dimensions of performance, including indicators across the range of structure, process and outcome and taking into account typical performance indicators for this care setting,^25^ which are likely to be used also in other long-term care systems. More detailed indicator descriptions are provided in table 1.

**Table 1:** List of outcom, process and structural indicators used in the study

Indicator	Numerator	Denominator	Indicator source
**Outcome indicators**			
Pressure ulcer rates of category 2 to 4 developed in the facility	Number of residents, who had at least one pressure ulcer of category 2 to 4 developed in the facility in 2019 x 100.	Residents days of care in 2019.	MeS Lab performance survey.
Rate of falls leading to ER visit, hospitalisation or death	Number of falls in 2019 that required emergency department visit, hospitalisation or resulted in death.	Residents days of care in 2019.	MeS Lab performance survey.
Rate of restraints use other than bed rails	Number of residents on whom physical restraints other than full-length bed rails were applied.	Number of residents who were cared for in the facility for at least 1 day in 2019.	MeS Lab performance survey.
Percentage of residents with a urinary tract infection	Number of residents, who had a urinary tract infection in 2019 x 100.	Residents days of care in 2019.	MeS Lab performance survey
Percentage of residents who reported pain above the threshold	Number of persons defined in the denominator, who had a pain valuation on a VAS or PAINAD scale higher than 3 in 2019 x 100.	Number of residents who resided in the facility for 45 days or more and had a least one pain valuation.	MeS Lab performance survey.
**Process indicators**			
Percentage of residents who received influenza vaccine	Number of persons defined in the denominator, who received an influenza vaccination.	Number of residents who were cared for in the facility for the entire period dedicated to the vaccination campaign.	MeS Lab performance survey.
Availability of a quality officer	Binary response, yes/no. Data refer to 2019.		MeS Lab mapping survey.
Quality certification of the facility with certificates ISO 9001 or UNI 10881	Binary response, yes/no Data refer to 2019		MeS Lab mapping survey.
Availability of administrative software	The use of administrative software is conceived in the survey as separate from electronic health record, software for accounting and software for billing. Binary response, yes/no. Data refer to 2019.		MeS Lab mapping survey.
Job satisfaction ratings	Sum of scores given by respondents to the question ‘I am satisfied with my job’.	Number of respondents to the question ‘I am satisfied with my job’.	MeS Lab organisational climate survey.
**Structural indicators**			
Number of beds in the facility	Integer. Data refer to fourth quarter of 2020.		ISS quarterly COVID-19 survey.
Healthcare workers per available bed	Number of healthcare workers in the facility reported as available in the fourth quarter 2020.	Number of beds in the facility reported as available in the fourth quarter 2020.	ISS quarterly COVID-19 survey.
Availability of an isolation area	Is there an area available to isolate COVID-19 positive residents? Binary response, yes/no. Data refer to the fourth quarter 2020.		ISS quarterly COVID-19 survey.

ER, emergency room; ISS, Italian National Institute for Health; MeS Lab, Management and Healthcare Laboratory ; PAINAD, Pain Assessment in Advanced Dementia; VAS, Visual Analigue Scale.

#### COVID-19 incidence and prevalence data

To assess the capacity to both prevent and contain COVID-19 outbreaks we considered the percentage of residents in isolation in a given week. We considered the number of resident in isolation as the best approximation of COVID-19 prevalence among residents, as the national rule at the time was to isolate all COVID-19 positive residents.^26^ The week with the highest percentage of residents in isolation was chosen as the reference value for each nursing home. The measure is used under the assumption that the value considered is indicative not only of the capacity to prevent COVID-19 from entering the facility, but also of the capacity to contain or slow the spread of the outbreak throughout the facility. We also considered whether there were COVID-19 cases reported among the personnel in any of the weekly data available for each facility.

#### Data analysis

The nursing homes were grouped into four groups: (1) nursing homes with no reported cases of COVID-19, (2) nursing homes that only reported cases among personnel, but not among residents, (3) nursing homes that reported less than 15% of residents in isolation in any 1 week and (4) nursing homes which reported 15% of residents in isolation or more in any 1 week. The threshold of 15% to separate groups 3 and 4 was chosen empirically, to minimise the number of small nursing homes with very few cases that might be categorised as large outbreaks and also minimise the number of large nursing homes with many cases that might be categorised as small outbreaks. We also performed a sensitivity analysis with the threshold set at 30% of residents in isolation (online supplemental file 2). We considered the potential protective effect of experiencing a COVID-19 outbreak during the first wave. Hence, we excluded all nursing homes that reported more than 15% of positive COVID-19 cases among residents during the first wave, from all groups except group 4.

10.1136/bmjoq-2023-002560.supp2 Supplementary data

The four nursing homes groups on one hand and the outcome, process and structural indicators on the other hand were the variables that we compared. For each indicator, we looked at correlations with the four outbreak groups using either bar charts or box plots and tested the statistical significance using the Kruskall-Wallis test for continuous variables or χ^2^ for binomial variables. Statistical significance was set at p value≤0.05. Statistical analysis was conducted using the software R V.2022.02.3.

### Qualitative phase

The purpose of the qualitative phase of the study was to better understand the findings of the quantitative phase. We organised a series of group discussions with nursing home managers and other staff members who were involved in the organisation of the facilities’ response to COVID-19.

A convenience sampling approach was used. In order to ensure a heterogeneity of experiences with COVID-19 outbreaks, we recruited nursing homes from all four of the outbreak groups created through the data analysis. We asked nursing homes to identify top managers or other persons involved in organisational decisions to tackle the pandemic. Nursing homes were recruited mostly by phone. They also received an email with the background and purpose of the study and of the group discussions. The quantitative findings were not made available prior to the group discussion.

#### Group discussions

All group discussions were moderated by the first author (MP), supported by at least one other researcher (EP and SB). Each group discussion was structured in seven modules, each dedicated to the discussion of one or a small group of indicators. The topics (and corresponding indicators) of the modules were: outcome performance indicators, availability of a quality officer, quality certification of the facility, job satisfaction ratings, availability of administrative software, staffing levels and the availability of a COVID-19 isolation area. Facility size as a measure on which nursing homes largely have no influence, was not discussed in a separate module. Within each module, the discussion was prompted by a live poll of participants on whether in their experience each indicator or group of indicators to be discussed played a role in the capacity to prevent and contain COVID-19 outbreaks in their facilities. The poll was followed by a discussion centred on the polling question. The results of the quantitative analysis were shown to the participants after an initial discussion, also providing an opportunity for additional comments. Each group discussion lasted about 2 hours and was conducted in Italian. All the interviews were recorded and transcribed verbatim.

#### Data analysis

The approach was low-inference and descriptive using a thematic analysis method. We used a set of themes, deductively determined, based on the seven topics that organised the group discussions. Within each theme, codes were assigned inductively. The first group discussion was coded by two authors (MP and SB) and compared. Based on this, one author (MP) coded the remaining transcripts. The findings were discussed among all the authors. The software QDA miner lite V.2.0.8 was used for the qualitative phase.

### Patient and public involvement

Patients were not involved in the study. The qualitative part of the study was aimed at capturing the perspective of nursing homes.

All participants were informed of the purpose of the study verbally and in writing. They had the opportunity to ask questions and gave oral and written consent to being interviewed for the purpose of the study and to recording of the group discussions. Verbatim transcripts were anonymised prior to data analysis to safeguard the confidentiality of participants.

## Results

### Quantitative phase

Our database comprised of 159 nursing homes that participated in both the Italian National Institute for Health surveys and the MeS Lab surveys (table 2). There were 319 registered nursing homes in Tuscany as of May 2020,^24^ so our database includes 50% of them. Among the included nursing homes there were 50 in group 1 (no COVID-19 cases in the reported period), 26 in group 2 (only cases among personnel), 40 in group 3 (smaller outbreak among residents) and 43 in group 4 (larger outbreaks).

**Table 2:** Basic characteristics of nursing homes in Tuscany included in the analysis

Data description	Median	IQR (25%–75%)	Minimum and maximum values
Number of authorised beds on 31 December 2019	42 beds	30–60	10–225
Number of residents on 31 December 2019	40.5 residents	28–59	10–220
Share of non-self-sufficient residents on 31 December 2019	94%		
Number of personnel on 1 October 2020	30.5 personnel	23–42	9–135

Only a subset of these nursing homes took part in the healthcare performance survey and in the survey on personnel experiences, where participation is voluntary. For this reason, the number of nursing homes in each indicator calculation varies.

Table 3 shows the results of statistical testing of the relationship between outbreak groups and the selected indicators. The relationship is statistically significant in three cases: reporting pain above threshold, use of administrative software and number of beds. These relationships are represented graphically in online supplemental file 3. Higher levels of the indicator of pain above the threshold and the number of beds were observed in the two groups of nursing homes, where there were cases among residents. Notably, an administrative software is more often in use among the nursing homes that experienced outbreaks among residents.

10.1136/bmjoq-2023-002560.supp3 Supplementary data

**Table 3:** Result of statistica testing of the relationship between outbreaks groups of nursing homes and selected indicators

Indicator	No. of included nursing homes	Test statistic (χ^2^ value)	P value
Outcome indicators			
Pressure ulcer rates of category 2 to 4 developed in the facility	48	0.32	0.955
Rate of falls leading to ER visit, hospitalisation or death	48	3.18	0.365
Rate of restraints use other than bed rails	49	2.90	0.407
Percentage of residents with a urinary tract infection	48	5.21	0.157
Percentage of residents who reported pain above the threshold	45	9.93	0.019*
Process indicators			
Percentage of residents who received influenza vaccine	48	1.62	0.655
Job satisfaction ratings	32	0.956	0.309
Availability of a quality officer	151	2.39	0.496
Quality certification of the facility with ISO 9001 or UNI 10881	157	5.92	0.116
Availability of administrative software	151	9.64	0.023*
Structural indicators			
Number of beds in the facility	155	15.12	0.002*
Healthcare workers per available bed	155	0.76	0.858
Availability of a COVID-19 isolation area	158	5.88	0.117

*Statistically significant at P value < 0,05.

ER, emergency room.

### Qualitative phase

Four group discussions took place between 23 March and 21 April 2022. Between 4 and 7 nursing homes participated in each group discussion, totalling 22 nursing homes involved. The participating nursing homes’ sizes varied between 18 and 151 beds; four nursing homes belonged to outbreak group 1, five to outbreak group 2, seven to outbreak group 3 and six nursing homes belonged to outbreak group 4.

Between 1 and 3 persons from each nursing home took part in the discussion, for a total of 29 participants (between 7 and 8 in each group discussion). Among the participants, 16 were top managers (the positions’ names vary depending on the organisational structure), 5 were head nurses or held similar positions and the rest held different roles of responsibility within the facility.

The main findings related to each discussion module were grouped into possible explanations for the absence and possible explanations for the existence of an observable relationship between the indicator or the group of indicators and the capacity to prevent and contain COVID-19 outbreaks in the facility (table 4). Examples of participants’ statements supporting each finding are presented in online supplemental file 4.

10.1136/bmjoq-2023-002560.supp4 Supplementary data

**Table 4:** Main findings on the relationship between selected indicators and capacity to prevent and contain COVID-19 outbreaks in nursing homes

Discussion module	Possible explanation for the existence of an observable relationship	Possible explanation for the absence of an observable
Outcome performance indicators		
	Working on outcome performance indicators strengthens experiences with establishing and implementing protocols and procedures.	COVID-19 was a new challenge for which work on outcome performance indicators did not prepare.
	Working on outcome performance indicators strengthens experiences with on-the-job training.	
	Working on outcome performance indicators encourages a proactive approach to risk management.	
	Good outcome performance indicators may imply more attention to infection prevention prior to COVID-19.	
	Good outcome performance indicators may imply less need for visits to emergency departments and hospitals, hence reducing COVID-19 exposure risk.	
Availability of a quality officer		
	Often quality officers translate national and regional COVID-19-related guidelines and requirements into facility-level protocols and procedures.	If there is no quality officer, somebody else would perform the role of translating COVID-19-related guidelines and requirements.
	Quality officers might have a different perspective than general management or administrative staff when establishing new protocols and procedures.	There is no relation, because once a case is recognised, it is already too late for any intervention to have a meaningful effect.
Quality certification of the facility with ISO 9001 or UNI 10881		
	Helpful to the extent that contingency plans were required and prepared because of certification requirements.	The requirements of any certification system did not foresee COVID-19, so there were no related requirements.
Availability of administrative software		
	If an electronic health record is part of an administrative software, then the record indeed helps.	A software does not help in COVID-19-related activities.
	Perhaps facilities that do not have an administrative software were less accurate in reporting data.	
	Correlation between size and availability of software.	
Job satisfaction ratings		
	Good relationships with management and within work teams likely means easier and quicker uptake of new procedures.	Dedication of the staff was essential, but this is not the same as satisfaction.
		The pandemic created a sense of responsibility among staff independent of job satisfaction.
		The pandemic created a sense of unity in the face of adversity independent of job satisfaction.
		High turnover of staff may indicate that 2019 data are not really relevant.
Healthcare workers per available bed		
	More personnel allows more extensive precautionary measures.	Number of personnel does not capture the dedication of staff.
	The establishment of ‘staff bubble’ often requires a lot of personnel.	Staff turnover is a problem because of the need for training and integration in work processes.
		Unclear testing rules for temporary personnel.
		Once personnel is infected, you have many absences.
Availability of an isolation area		
	The isolation area was used for quarantine.	Having an isolation room is not sufficient to isolate residents, the facility’s architecture is often a limiting factor.
		Strict isolation is not really feasible in a nursing home, with or without an isolation room.
		Once a case is identified the virus has already spread in the facility.

#### Outcome performance indicators

Participants identified a number of indirect potential pathways through which good results on the outcome performance indicators might be conducive to improved capacity to prevent and contain COVID-19 outbreaks. Those mostly derive from the assumption that good results on these indicators imply intense work on clinical risk management, design and implementation of protocols and procedures, including staff training. These activities create a skill set in risk management that was helpful also in tackling the COVID-19 pandemic.

On the other hand, a number of participants thought that there is no relation between results on these indicators and COVID-19 outbreaks, as the issues are different and working on outcome performance indicators did not prepare the organisation to tackle the new challenges posed by the pandemic.

#### Availability of a quality officer

Participants emphasised the important role of translating COVID-19-related guidelines and requirements that were often adopted by regional and national authorities, into internal protocols and procedures. This role was often covered by the quality officers, sometimes there was an entire task force and information and experiences were exchanged between nursing homes that are part of a larger corporation. However, some participants also indicated that in the absence of a quality officer, the role was covered by other staff members, hence it is not clear that the presence of a quality officer makes a difference. Furthermore, it is worth noticing that additional medical support of one full-time equivalent for every 300 residents was made available to nursing homes in the Tuscany region from May 2020.^27^

#### Quality certification of the facility with ISO 9001 or UNI 10881

In Tuscany, nursing homes must be accredited by the regional authority to be allowed to operate within the public health and social care network.^28^ Within this context, a number of nursing homes are also certified by other internationally recognised standards, with ISO 9001 and UNI 10881 being the most common. Participants agreed that there were no procedures required by any standard that they used, which addressed specifically the pandemic situation. Participants from one nursing home mentioned that there are many similarities between the procedures that they set-up due to COVID-19 and contingency plans that they had previously prepared in case of major disruptions (fire, earthquake, etc).

#### Availability of administrative software

Participants had difficulties in identifying potential reasons that might explain the association between the availability of an administrative software and COVID-19 outbreaks. An important hypothesis mentioned by participants is the correlation between the availability of an administrative software and facility size. One group hypothesised, that the data might be less accurate for those facilities that do not have such a software, resulting in an underestimation of cases. However, another group disagreed with that possibility. The discussions suggest that it might not be always clear what is intended by administrative software and how it differs from the medical health record, accounting and billing software, which are the other options in the survey question that provided the data.

#### Job satisfaction ratings

The participants’ positions varied considerably when asked about the potential impact of staff job satisfaction on the capacity to prevent and contain COVID-19 outbreaks. Participants rejected the hypothesis that dissatisfied staff would perform their duties in an unsafe way. In one group the moderator asked directly, if there might be a common cause for staff satisfaction and the capacity to prevent and contain COVID-19 outbreaks hidden in good management practices. Participants did not confirm the validity of this hypothesis. Teamwork was identified as key to successfully prevent and contain outbreaks. At the same time, participants also pointed out how teamwork strengthened in the face of the pandemic often regardless of job satisfaction prior to the pandemic.

#### Healthcare workers per available bed

Participants mostly agreed that personnel-related issues were essential to the prevention and containment of COVID-19 outbreaks in nursing homes. One participant expressed a dissenting view, indicating that the lack of personnel has influenced the quality of non-health-related services considerably, but the procedures to prevent and contain COVID-19 were always followed.

It is important to note that there have been minimum staff requirements established by the regional authority since before the pandemic.^29^ Participants indicated that as a consequence of COVID-19 the procedures for providing safe care were adapted, which increased the workload and hence the need for personnel. At the same time, there was a drain of personnel from nursing homes to hospitals, due to regional policies and different working conditions between nursing homes and other healthcare institutions.

#### Availability of an isolation area

Participants emphasised the importance of distinguishing between having a room where COVID-19 positive residents can be placed and being able to observe the isolation procedures necessary to prevent the spread of the infection. Participants mentioned the difficulties in establishing such procedures within nursing homes where the infrastructure is not planned for these purposes. Some participants also mentioned the valuable expert support provided by the district health authority for planning isolation procedures under such circumstances.

## Discussion

The quantitative analysis did not show a clear association between pre-existing quality management outcome and process measures, and COVID-19 in nursing homes. The association was statistically significant only with respect to the share of residents experiencing pain above the threshold and the availability of administrative software. Participants in the group discussions provided several potential explanations for the existence of an association between the quality management measures considered and COVID-19 outbreaks as well as for the absence of such an association. The indicators considered in this study were often seen as not related to activities undertaken to prevent and contain COVID-19 outbreaks in nursing homes. Participants also emphasised the extraordinary nature of the pandemic, which created a sense of unity and responsibility that allowed major changes in the organisation of nursing home facilities to take place quickly and effectively, regardless of pre-existing circumstances.

The association between the availability of an administrative software and COVID-19 outbreaks may be due to the confounding effect of size, associated with both COVID-19 outbreaks and availability of administrative software. However, we also cannot exclude the potential for reporting bias, whereby the availability of an administrative software might support more accurate reporting.^30 31^

The performance assessment of nursing homes in Tuscany does not include an overall quality rating, as is the case of nursing home compare in the USA.^32^ This lack of an overall quality rating prevents an analysis using a single overall measure of quality. However, consideration of a number of quality measures allows a more in-depth exploration of the potential association between quality and COVID-19 outbreaks. The lack of a statistically significant association between the pre-existing quality measures that we considered and COVID-19 outbreaks is in line with the findings of most papers on the issue.^8^

Our study added important contextual considerations to these findings. For example, the consideration of external certification of quality in Tuscany must be interpreted in the context of an obligatory accreditation system for nursing homes.^28^ This likely indicates that all nursing homes are experienced in quality management as this is part of the accreditation requirements, regardless of an additional external quality certification. Another example that points to the same caveat is staffing. In Tuscany, there are staffing requirements that all nursing homes must abide to,^29^ while participants explained that due to high costs, it is unlikely that these requirements are exceeded considerably. And indeed the quantitative analysis shows relatively little variation in staffing rates. The importance of contextual considerations when looking at the association between staffing rates and COVID-19 outbreaks has been noted also by other studies,^8 9 12^ although the specific considerations differ depending on the settings of the study.

The qualitative part of the study also revealed some limitations of the information provided by the variables considered. For example, the availability of a quality officer is not related to a well-defined role for the quality officer. Similarly, the availability of an isolation area for COVID-19-positive residents was open to different interpretations, as many nursing homes did not have the architectural design to be able to fully comply with isolation requirements, even if there was such a designated area. A recent study in the Italian region of Lazio that considered some of these architectural elements^33^ did not find a correlation with the risk of COVID-19 infection in nursing homes.

Several recent reports called for urgent quality improvements in nursing homes as a lesson learnt from the pandemic,^34–36^ regardless of the lack of convincing quantitative evidence on the association between quality and COVID-19 outbreaks in nursing homes. These reports emphasise the importance of appropriate staffing and architectural design considerations, which we considered structural indicators. However, these aspects are not captured sufficiently well by the indicators considered in this study. It is less clear whether we need a specific set of measures that would capture the preparedness of nursing homes to face such emergencies in the future. The introduction of such measures and their use as quality indicators imply a clear definition of the responsibilities of nursing homes in a future pandemic, in particular in relation to the responsibilities of hospitals, civil protection agencies, social service providers and other relevant actors.^37^ Only with such clarity it is possible to develop or identify indicators that will appropriately monitor the emergency preparedness and resilience of nursing homes.

In Italy, the National Recovery and Resilience Plan^38^ supports a reform of health and social care services, that focuses on closeness with the community and includes support for home care services and community care centres. In Tuscany, the set of indicators that providers of long-term care services have to report on has been updated,^39^ even though this was part of a broader reform process initiated prior to the pandemic. Our study also reinforces the need for caution when seeking overall measures of nursing homes’ quality, as there is a risk of overlooking important dimensions on which these measures are constructed.

### Strengths and limitations

The focus of the study on the period of autumn 2020 is of particular interest as it captures the circumstances before vaccination was available, but after the shock of the first wave, when stakeholders had some time to build a knowledge base about the virus and prepare their responses. The lack of weekly data on COVID-19 in nursing homes throughout the first wave of the pandemic is a limitation of our data set. However, with the aggregated data available to us we were able to minimise the effects of outbreaks in specific nursing homes during the first wave on potential outbreaks during the second wave. By looking at different aspects of quality through a number of structural, process and outcome measures, the study offers a more nuanced look at the potential relationship between quality management and COVID-19 outbreaks. A limitation is represented by the lack of adjustment for factors such as the size of the facilities considered, the prevalence of COVID-19 in the surrounding community or the health status of residents. The choice was dictated by a relatively small sample size, the availability of aggregated data and the wish to do a basic exploration of associations between the selected variables. A further limitation is the inability to consider and control for specific infection prevention procedures, audits or other actions, as these data were not collected systematically prior to the pandemic. The interpretation of the data was strongly enhanced by the qualitative part of the study. The study is limited to one Italian region. Consequently, the experiences of participants were more homogeneous and the local specifics of the long/term care system could be taken into account. However, this limits the generalisability of the findings.

## Conclusions

Our quantitative exploration of the association between several pre-existing quality management measures and COVID-19 outbreaks in nursing homes showed statistically significant associations with 3 out of 12 indicators considered. While it is worth investigating this further, the findings do not provide strong support for an association between pre-existing quality management in the facility and the capacity to prevent and contain COVID-19 outbreaks.

Similarly in the qualitative part of the study, the discussions with nursing home managers revealed that none of the measures explored was particularly good at exposing the challenges related to preventing and containing COVID-19 outbreaks.

If nursing homes are expected to be prepared for a future pandemic, further studies are necessary to identify the indicators best suited to monitor their emergency preparedness, and ability to absorb shocks, adapt to changes and maintain essential functions during crises.

## Data Availability

Data are available upon reasonable request. Quantitative data that support the findings of this study are available from the corresponding author, MP, upon reasonable request. The qualitative data supporting the findings of this study are available within the article and its supplementary materials.

## References

[1] Bezzini D , Schiavetti I , Manacorda T , et al . First wave of COVID-19 pandemic in Italy: data and evidence. In: Asea AAA , Kaur P , eds. Coronavirus therapeutics – volume II: clinical management and public health. Cham: Springer International Publishing, 2021: 91–113. 10.1007/978-3-030-85113-2_6 35137370

[2] Scortichini M , Schneider Dos Santos R , De’ Donato F , et al . Excess mortality during the COVID-19 outbreak in Italy: a two-stage interrupted time-series analysis. Int J Epidemiol 2021;49:1909–17. 10.1093/ije/dyaa169 33053172 PMC7665549

[3] Notarnicola E , Perobelli E , Rotolo A , et al . Lessons learned from Italian nursing homes during the COVID-19 outbreak: a tale of long-term care fragility and policy failure. J Long Term Care 2021;221–9. 10.31389/jltc.73

[4] Gabutti G , d’Anchera E , De Motoli F , et al . The epidemiological characteristics of the COVID-19 pandemic in Europe: focus on Italy. Int J Environ Res Public Health 2021;18:2942. 10.3390/ijerph18062942 33805624 PMC8000566

[5] León M , Arlotti M , Palomera D , et al . Trapped in a blind spot: the COVID-19 crisis in nursing homes in Italy and Spain. Soc Policy Soc 2021;1–20. 10.1017/s147474642100066x

[6] Lombardo FL , Bacigalupo I , Salvi E , et al . The Italian national survey on Coronavirus disease 2019 epidemic spread in nursing homes. Int J Geriatr Psychiatry 2021;36:873–82. 10.1002/gps.5487 33368636 PMC8247061

[7] ARS Toscana . Welfare E salute in toscana 2021 [welfare and health in tuscany 2021]. ARS Toscana; 2021. Available: https://www.ars.toscana.it/images/pubblicazioni/Collana_ARS/2021/welfare_e_salute/Libro_WS_1_def.pdf

[8] Konetzka RT , White EM , Pralea A , et al . A systematic review of long-term care facility characteristics associated with COVID-19 outcomes. J Am Geriatr Soc 2021;69:2766–77. 10.1111/jgs.17434 34549415 PMC8631348

[9] Dykgraaf SH , Matenge S , Desborough J , et al . Protecting nursing homes and long-term care facilities from COVID-19: a rapid review of international evidence. J Am Med Dir Assoc 2021;22:1969–88. 10.1016/j.jamda.2021.07.027 34428466 PMC8328566

[10] Dyer AH , Fallon A , Noonan C , et al . Managing the impact of COVID-19 in nursing homes and long-term care facilities: an update. J Am Med Dir Assoc 2022;23:1590–602. 10.1016/j.jamda.2022.06.028 35922016 PMC9250924

[11] Liljas AEM , Morath LP , Burström B , et al . The impact of organisational characteristics of staff and facility on infectious disease outbreaks in care homes: a systematic review. BMC Health Serv Res 2022;22:339. 10.1186/s12913-022-07481-w 35291990 PMC8921437

[12] Shallcross L , Burke D , Abbott O , et al . Factors associated with SARS-Cov-2 infection and outbreaks in long-term care facilities in England: a national cross-sectional survey. Lancet Healthy Longev 2021;2:e129–42. 10.1016/S2666-7568(20)30065-9 33655236 PMC7906733

[13] Romero-Ortuño R , Kennelly SP . COVID-19 deaths in Irish nursing homes: exploring variation and association with the adherence to national regulatory quality standards. 2020. Available: https://ltccovid.org/wp-content/uploads/2020/06/Ireland-care-home-variations-in-numbers-of-deaths-and-quality-indicators.pdf

[14] Tamara Konetzka R , Yan K , Werner RM . Two decades of nursing home compare: what have we learned? Med Care Res Rev 2021;78:295–310. 10.1177/1077558720931652 32538264

[15] Cohen-Mansfield J . The impact of COVID-19 on long-term care facilities and their staff in Israel: results from a mixed methods study. J Nurs Manag 2022;30:2470–8. 10.1111/jonm.13667 35538706 PMC9348504

[16] Thomas M , Frazier E , Garr D . Managing COVID-19 transmission in long-term care: a qualitative study of high performing facilities. Am J Infect Control 2023;51:234–7. 10.1016/j.ajic.2022.06.023 35839959 PMC9284584

[17] European Commission Directorate-General for Employment Social Affairs and Inclusion . Long-term care report: trends, challenges and opportunities in an ageing society. Volume II, country profiles. Luxembourg Publications Office of the European Union; 2021.

[18] Emergenza epidemiologica Covid.19: linee di indirizzo alle strutture residenziali sociosanitarie (RSA, RSD, ecc.) per le modalità di accesso dei familiari fino al termine delle misure straordinarie sul COVID-19. [Covid.19 epidemiological emergency: guidelines for residential social-health facilities (RSA, RSD, etc.) for methods of access for family members until the end of the extraordinary measures on COVID-19]. 2020. Available: https://www.regione.toscana.it/documents/10180/24263283/Linee+di+indirizzo.pdf/175e9a32-a5f5-46e2-6448-5cb64188a86b?t=1591867493258

[19] Ivankova NV , Creswell JW , Stick SL . Using mixed-methods sequential explanatory design: from theory to practice. Field Methods 2006;18:3–20. 10.1177/1525822X05282260

[20] Lee S-YD , Iott B , Banaszak-Holl J , et al . Application of mixed methods in health services management research: a systematic review. Med Care Res Rev 2022;79:331–44. 10.1177/10775587211030393 34253078

[21] Barsanti S , Pardini F , Colombini G , et al . Le Indagini di soddisfazione degli operatori delle residenze sanitarie assistenziali in toscana report 2019 [the satisfaction surveys of care workers in care homes in Tuscany 2019 report]. Pisa Tipografia Editrice Pisana Snc; 2020.

[22] Barsanti S , Pardini E , Colombini G , et al . Il Sistema di valutazione della performance delle RSA in regione toscana report 2019 [the performance evaluation system of care homes in the tuscany region 2019 report]. Pisa Tipografia Editrice Pisana snc; 2020.

[23] Barsanti S , Walker K , Seghieri C , et al . Consistency of priorities for quality improvement for nursing homes in Italy and Canada: a comparison of optimization models of resident satisfaction. Health Policy 2017;121:862–9. 10.1016/j.healthpol.2017.06.004 28687182

[24] National Institute for Health . Survey nazionale sul contagio COVID-19 nelle strutture residenziali e sociosanitarie, epidemia COVID-19, aggiornamento nazionale: 05 maggio 2020. [National survey on Covid 19 infections in long-term care facilities, Covid 19 epidemic, national update: 5 May 2020]. 2020. Available: https://www.epicentro.iss.it/coronavirus/pdf/sars-cov-2-survey-rsa-rapporto-finale.pdf

[25] Organization for Economic Cooperation and Development, European Commission . A good life in old age? Monitoring and improving quality in long-term care. OECD Publishing; 2013.

[26] ISS working group for infection prevention and control - Covid 19. Indicazioni ad interim per la prevenzione E Il controllo dell’Infezione DA SARS-COV-2 in strutture residenziali sociosanitarie [interim indications for the prevention and control of SARS-COV-2 infection in residential healthcare facilities, contract no: N.4/ 2020 Rev. Rome Istituto Superiore di Sanita; 2020.

[27] Order of the president of the regional council, 49. 2020. Available: https://www301.regione.toscana.it/bancadati/atti/Contenuto.xml?id=5251284&nomeFile=Ordinanza_del_Presidente_n.49_del_03-05-2020

[28] Regional act accreditation of facilities and personal services of the integrated social care system, 82. 2009. Available: http://raccoltanormativa.consiglio.regione.toscana.it/articolo?urndoc=urn:nir:regione.toscana:legge:2009-12-28;82

[29] Decree of the president of the regional executive of 9 January 2018 no. 2/R: provisions to implement article 62 of regional act no 41 of 24 February 2005. 2018. Available: https://inapp.org/sites/default/files/NORMATIVA/2018/Regionale/TO_Decreto%20n.2-R%20del%2009-01-18.pdf

[30] Schnelle JF , Bates-Jensen BM , Chu L , et al . Accuracy of nursing home medical record information about care-process delivery: implications for staff management and improvement. J Am Geriatr Soc 2004;52:1378–83. 10.1111/j.1532-5415.2004.52372.x 15271130

[31] Ancker JS , Shih S , Singh MP , et al . Root causes underlying challenges to secondary use of data. AMIA Annu Symp Proc 2011;2011:57–62. 22195055 PMC3243274

[32] Williams CS , Zheng Q , White AJ , et al . The association of nursing home quality ratings and spread of COVID-19. J Am Geriatr Soc 2021;69:2070–8. 10.1111/jgs.17309 34058015 PMC8242717

[33] Orlando S , Mazhari T , Abbondanzieri A , et al . Characteristics of nursing homes and early preventive measures associated with risk of infection from COVID-19 in Lazio region, Italy: a retrospective case-control study. BMJ Open 2022;12:e061784. 10.1136/bmjopen-2022-061784 PMC9170802 35667726

[34] European Commission Directorate-General for Employment Social Affairs and Inclusion . Long-term care report: trends, challenges and opportunities in an ageing society. Volume I. Luxembourg: Publications Office of the European Union, 2021.

[35] National Academies of Sciences Engineering and Medicine . The national imperative to improve nursing home quality: honoring our commitment to residents, families, and staff. Washington, DC: The National Academies Press, 2022:604. 36198022

[36] Marrocco FN , Coke A , Kitts J . Ontario’s long-term care COVID-19 commission final report. 2021. Available: https://files.ontario.ca/mltc-ltcc-final-report-en-2021-04-30.pdf

[37] Thomas S , Bolsewicz K , Latta R , et al . The impact of public health restrictions in residential aged care on residents, families, and staff during COVID-19: getting the balance right. J Aging Soc Policy 2022;1–20. 10.1080/08959420.2022.2110802 35946918

[38] National recovery and resilience plan. Italy tomorrow; 2021. Available: https://italiadomani.gov.it/it/home.html

[39] Resolution of the regional executive no. 245 of 13 March 2021 and following. 2021. Available: http://www301.regione.toscana.it/bancadati/atti/DettaglioAttiG.xml?codprat=2021DG00000000311

