# Supplementary material for: Relation between pre-existing quality management measures and prevention and containment of COVID-19 outbreaks in 159 nursing homes in Tuscany: a mixed methods study
Source: BMJ Open Qual. 2024 Apr 30;13(2):e002560. doi: 10.1136/bmjoq-2023-002560 (PMC11086181; doi:10.1136/bmjoq-2023-002560)
Supplement: Supplementary data [file bmjoq-2023-002560supp001.pdf]

Supplement 1

Checklist for mixed methods research manuscript preparation and review by Lee et al. (2022)*

Checklist Item	Manuscript reference
**Rational and description of MMRdesign**	
Provide a clear statement of the study purpose	Explained in the last paragraph of the Introduction section.
Explicitly describe the MMR design in accordance with Creswell's (2015) typology and use a diagram to illustrate the relationship and sequence of qualitative and quantitative research components	Described in the first paragraph of the Methods section and in Figure 1.
Justify why the MMR design is appropriate for meeting the study purpose	Explained in the first paragraph of the Methods section.
**Transparency in describing method details**	
Describe the study population(s) and sample(s); e.g., who, what, how many)	For the quantitative part, see the Data sources paragraph of the section Methods, subsection Quantitative phase and in the Results section, subsection Quantitative phase. For the qualitative part, see second paragraph of the Methods section, subsection Qualitative phase.
Describe the sampling procedures (including inclusion and exclusion criteria, recruitment)	For the qualitative part, see second paragraph of the Methods section, subsection Qualitative phase. For the quantitative part, data were not collected within this study. Data collection details are reported in the References cited in the Data sources paragraph of the Methods section, subsection Quantitative phase.
Describe qualitative data collection processes (how often data were collected, who collected the data, what kind of data collection instruments were used, how data were recorded—e.g., notes, transcripts)	Described under "Group discussion" of the Methods section, subsection Qualitative phase.
Describe quantitative data collection processes (how often data were collected, who collected the data, what kind of data collection instruments were used measurements, validity/reliability)	The quantitative data were not collected within this study. Data collection details are reported in the References cited in the Data sources paragraph of the Methods section, subsection Quantitative phase.
Describe qualitative data analysis processes (coding, single or multiple coders, replication logic, credibility)	Presented under "Data analysis" of the Methods section, subsection Qualitative phase.
Describe quantitative data analysis procedures (missing data and how they are handled, statistical tests used)	The exclusion of nursing homes, for which insufficient data was available, is described under "Data sources" of the section Methods, subsection Quantitative phase. The exclusion of nursing homes based on

	performance indicator data availability is described in the second paragraph of the Results section, subsection Quantitative phase. Data analysis is described under "Covid-19 incidence and prevalence data" and "Data analysis" of the Methods section, subsection Quantitative phase
**Integration of qualitative and quantitative research components**	
Interpret qualitative analysis results with appropriate quotes if necessary	The main findings are reported in Table 4, the supporting quotes are reported in Supplement 4.
Interpret quantitative analysis results in consideration of statistical significance, selection bias, and threats to validity	Potential caveats to quantitative data interpretation are discussed throughout the Discussion section.
Compare qualitative and quantitative results	The sequential explanatory study design involved attempts to interpret the quantitative data through a qualitative approach.
Address divergencies and inconsistencies between qualitative and quantitative results	Figure 4 provides potential explanations for associations as well as lack of associations between Covid-19 outbreaks and performance indicators as they emerged through the qualitative phase of the study. The implications are considered in the Discussion section.

*Lee SD, Iott B, Banaszak-Holl J, Shih SF, Raj M, Johnson KE, Kiessling K, Moore-Petinak N. Application of Mixed Methods in Health Services Management Research: A Systematic Review. Med Care Res Rev. 2022 Jun;79(3):331-344. doi: 10.1177/10775587211030393
