# Supplementary material for: Relation between pre-existing quality management measures and prevention and containment of COVID-19 outbreaks in 159 nursing homes in Tuscany: a mixed methods study
Source: BMJ Open Qual. 2024 Apr 30;13(2):e002560. doi: 10.1136/bmjoq-2023-002560 (PMC11086181; doi:10.1136/bmjoq-2023-002560)
Supplement: Supplementary data [file bmjoq-2023-002560supp002.pdf]

Supplement 2

Sensitivity analysis

Result of statistical testing of the relationship between outbreaks groups of nursing homes and selected indicators with threshold value of 30% of Covid positive residents separating outbreak groups 3 and 4.

Indicator	No. of included nursing homes	Test statistic (Chi square value)	p-value
**Outcome indicators**			
Nursing homes' pressure ulcer rates of category 2 to 4 developed in the facility	48	0,21	0,976
Nursing homes' rate of falls leading to ER visit, hospitalization or death	48	0,67	0,880
Nursing homes' rate of restraints use other than bed rails	49	3,78	0,287
Nursing homes' percentage of residents with a urinary tract infection	48	4,37	0,224
Nursing homes' percentage of residents who reported pain above the threshold	45	10,50	0,015*
**Process indicators**			
Nursing homes' percentage of residents who received influenza vaccine	48	1,84	0,607
Nursing homes' job satisfaction ratings	32	1,84	0,607
Availability of a quality officer	151	1.64	0.649
Quality certification of the facility with ISO 9001 or UNI 10881	157	8.42	0.038*
Availability of administrative software	151	10.22	0.017*
**Structural indicators**			
Number of beds in the facility	155	15,33	0,002*
Healthcare workers per available bed	155	0,75	0,860
Availability of a Covid 19 isolation area	158	4.44	0.217

*p-value < 0,05
