# Supplementary material for: Relation between pre-existing quality management measures and prevention and containment of COVID-19 outbreaks in 159 nursing homes in Tuscany: a mixed methods study
Source: BMJ Open Qual. 2024 Apr 30;13(2):e002560. doi: 10.1136/bmjoq-2023-002560 (PMC11086181; doi:10.1136/bmjoq-2023-002560)

Supplement 3

Box plot and bar charts of selected nursing homes' indicators by outbreak group

Figure 1: Box plot of pressure ulcer rates od category 2 to 4 developed in the facility in 2019 and nursing homes outbreak groups.

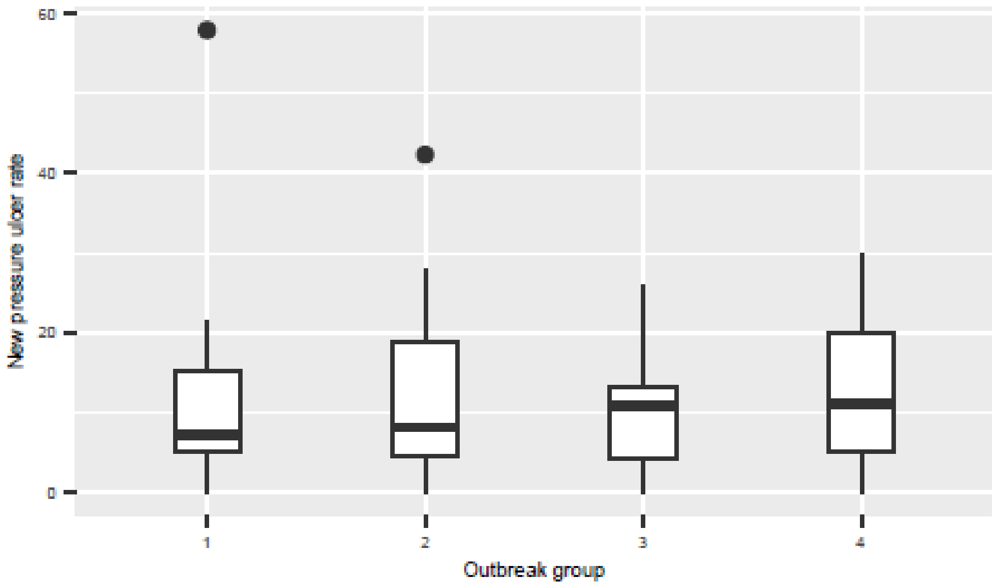

Figure 2: Box plot of nursing homes' rate of falls leading to emergency department visit, hospitalization or death in 2019 and nursing homes outbreak groups.

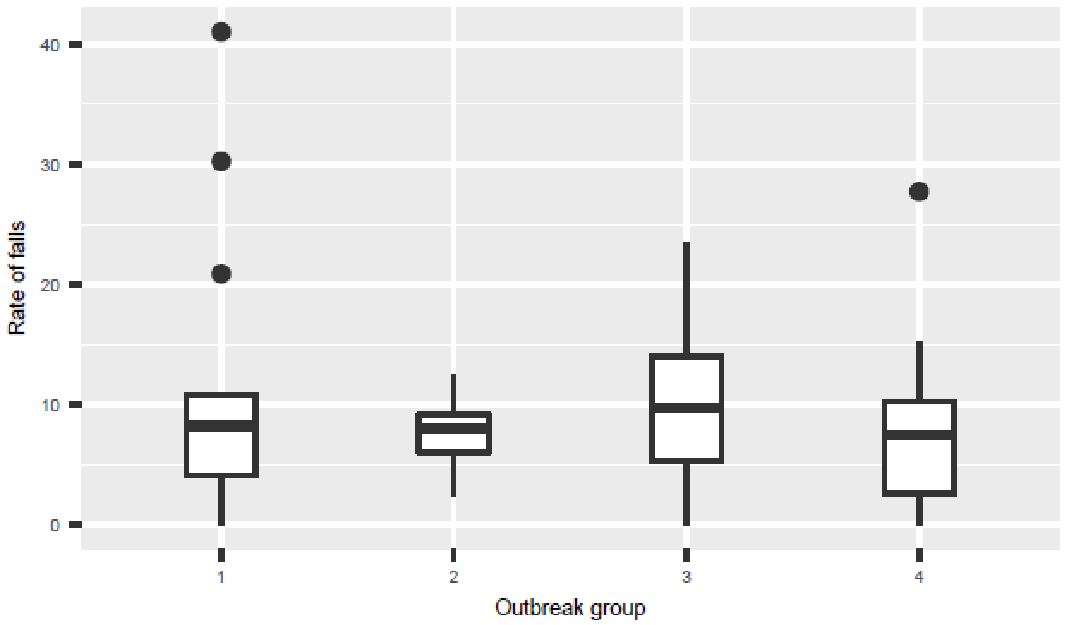

Figure 3: Box plot of nursing homes' rate of restraints use other than bed rails in 2019 and nursing homes outbreak groups.

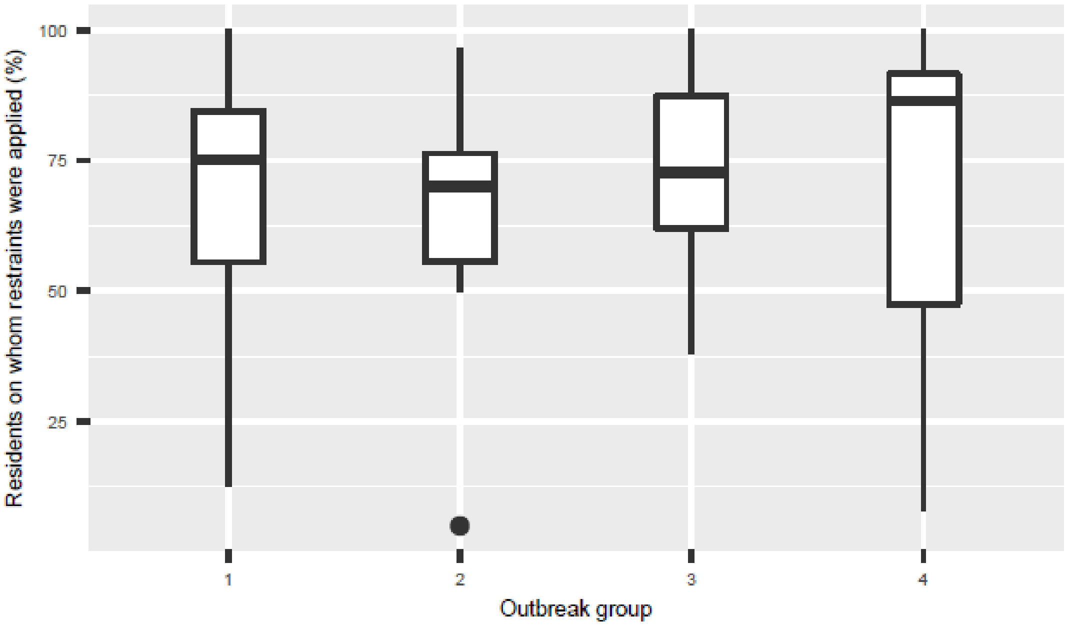

Figure 4: Box plot of nursing homes' percentage of residents with a urinary tract infection in 2019 and nursing homes outbreak groups.

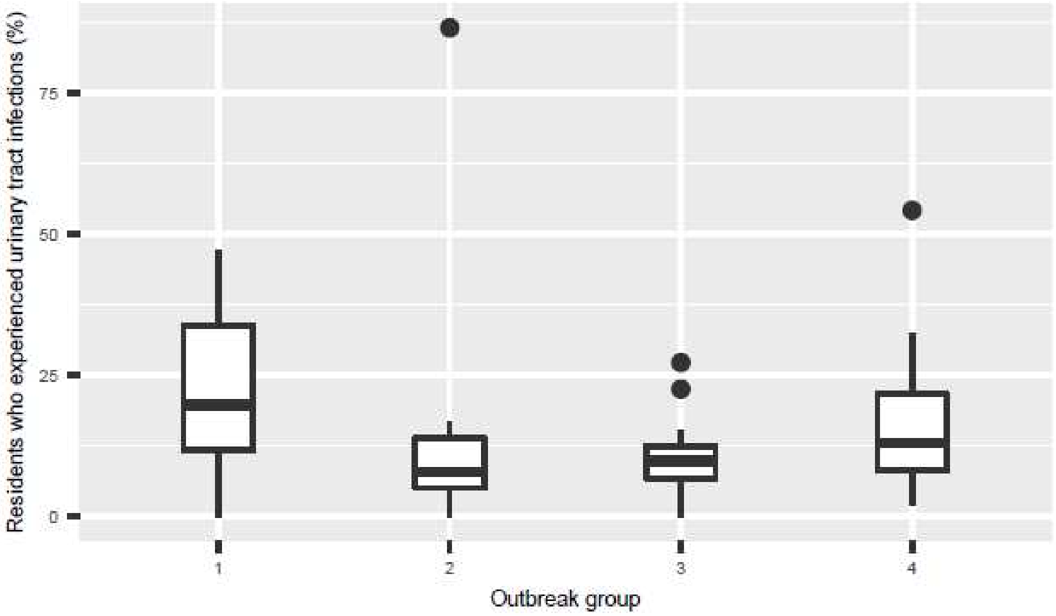

Figure 5: Box plot of nursing homes' percentage of residents who reported pain above the threshold in 2019 and nursing homes outbreak groups..

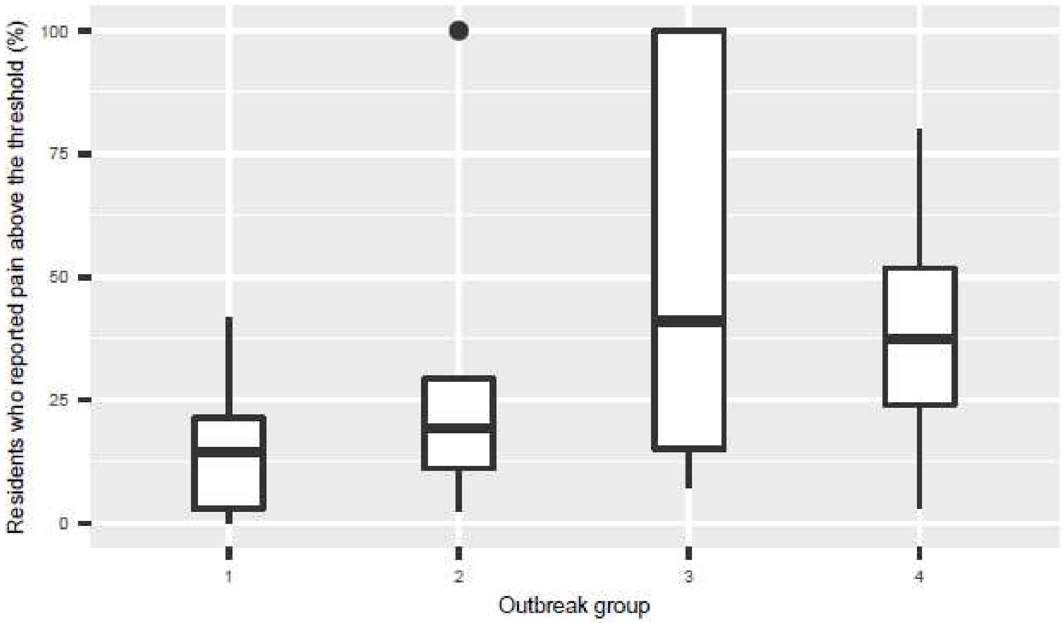

Figure 6: Box plot of nursing homes' percentage of residents who received the influenza vaccine and nursing homes outbreak groups.

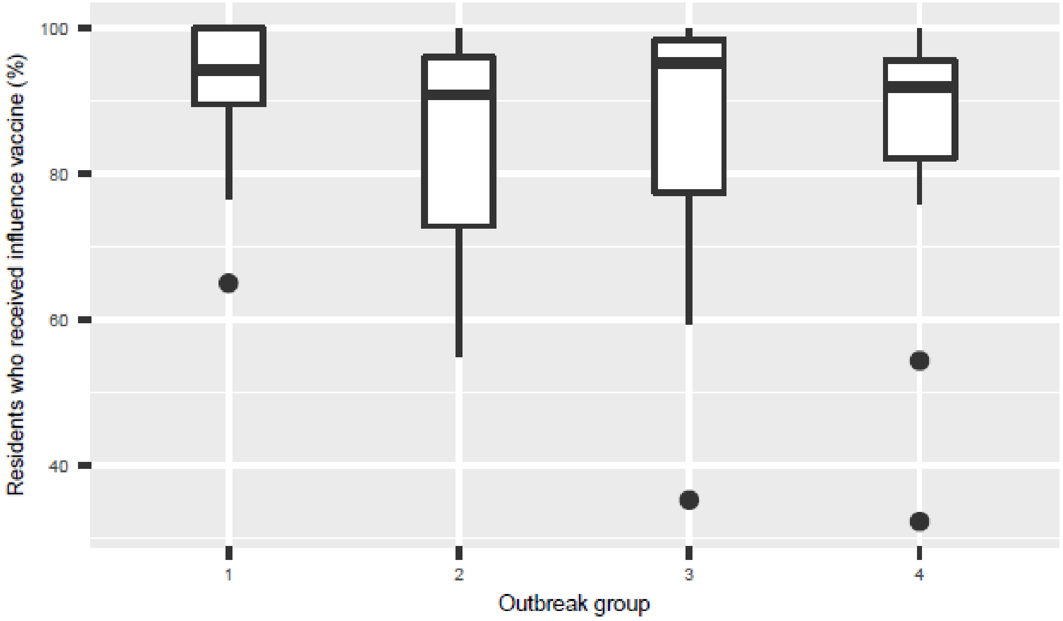

Figure 7: Box plot of nursing homes' job satisfaction rates in 2019 and nursing homes outbreak groups.

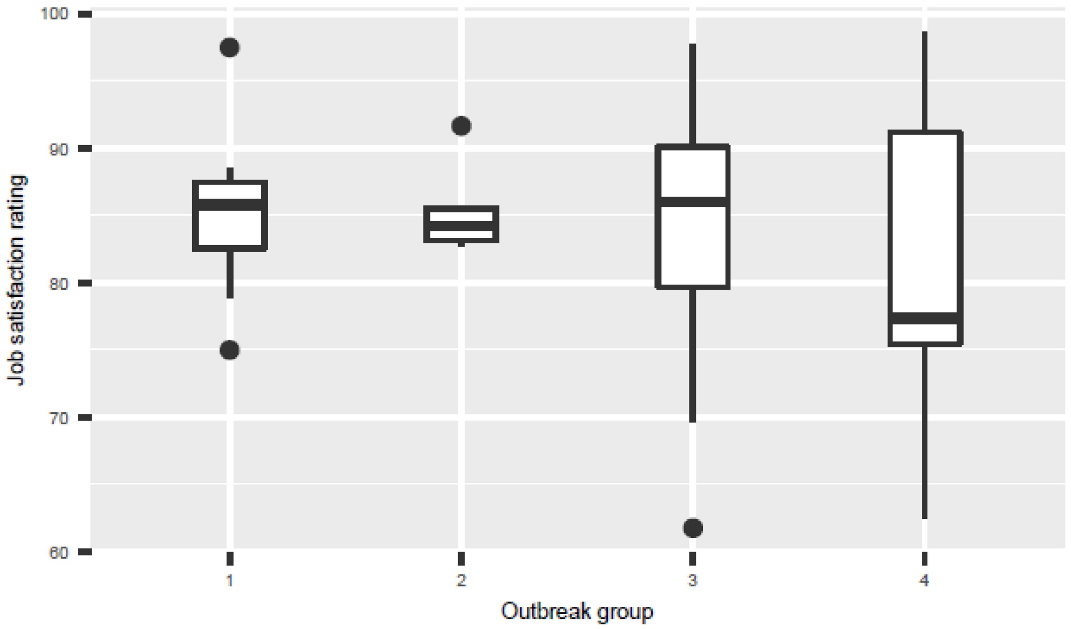

Figure 8: Percentage of nursing homes with an appointed quality officer in 2019 by nursing homes outbreak groups.

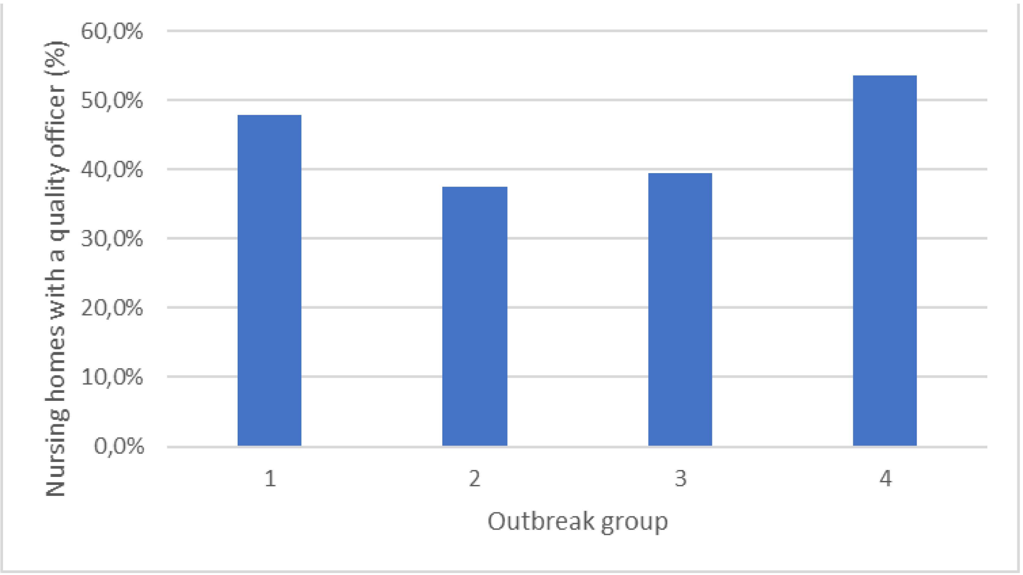

Figure 9: Percentage of nursing homes with a quality certificate (ISO 9001 or UNI 10881) in 2019 by nursing homes outbreak groups.

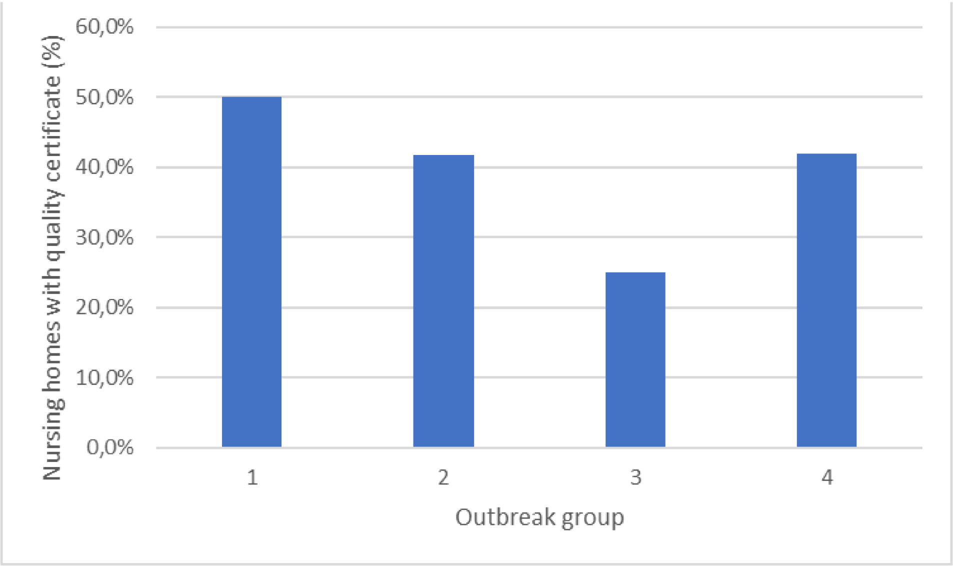

Figure 10:Percentage of nursing homes with an administrative software in 2019 by nursing homes outbreak groups.

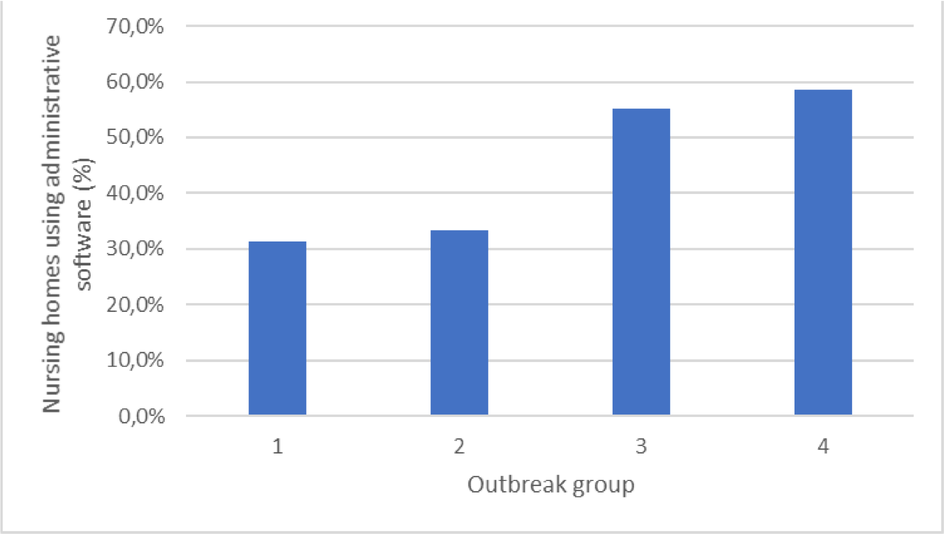

Figure 11: Box plot of nursing homes' number of beds and nursing homes outbreak groups.

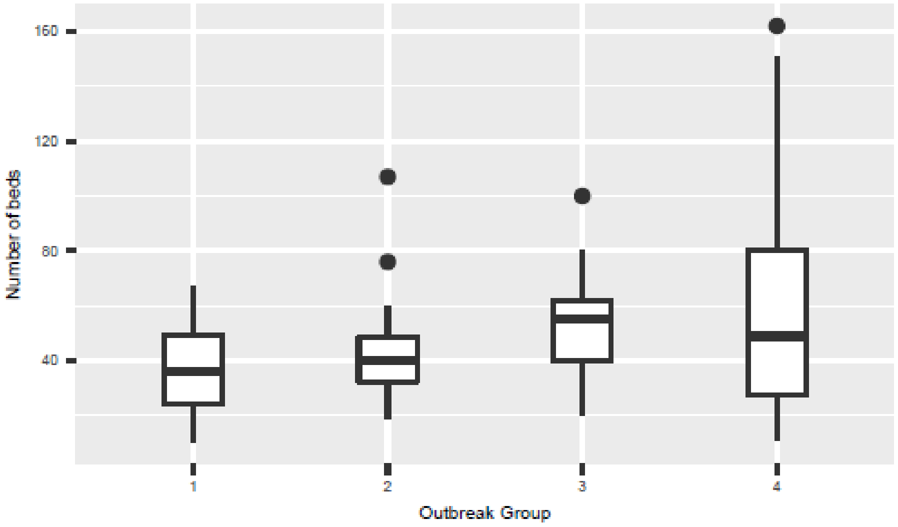

Figure 12: Box plot of nursing homes' healthcare workers per available bed in 2019 and nursing homes outbreak groups.

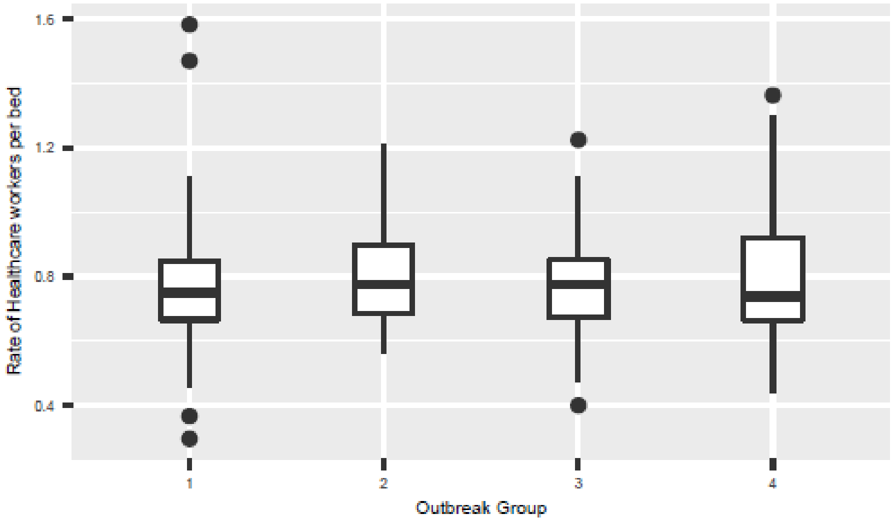

Figure 13: Percentage of nursing homes with an isolation area by nursing homes outbreak groups

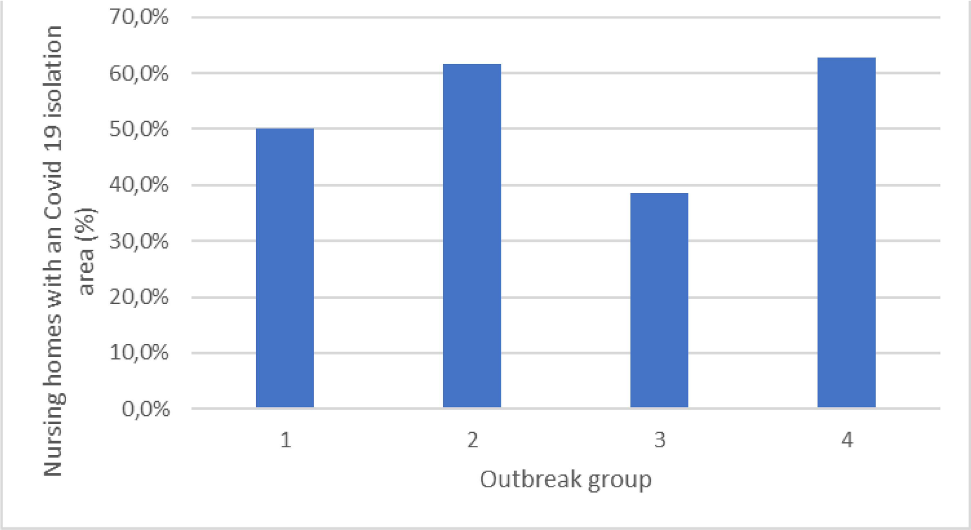

Supplement: Supplementary data [file bmjoq-2023-002560supp003.pdf]
