# Supplementary material for: Relation between pre-existing quality management measures and prevention and containment of COVID-19 outbreaks in 159 nursing homes in Tuscany: a mixed methods study
Source: BMJ Open Qual. 2024 Apr 30;13(2):e002560. doi: 10.1136/bmjoq-2023-002560 (PMC11086181; doi:10.1136/bmjoq-2023-002560)
Supplement: Supplementary data [file bmjoq-2023-002560supp004.pdf]

## Supplement 4

**Examples of participants' statments supporting the findings from the group discussions**

| | Finding | Example (quotation)* |
|---|---|---|
| **Outcome performance indicators** | | |
| *Possible explanation for the existence of an observable relationship* | | |
| | Working on outcome performance indicators strengthens experiences with establishing and implementing protocols and procedures. | ‘So, in my opinion, working with protocols and procedures, examining some processes or some care problems, such as pressure ulcers or infections, perhaps had already trained us for what we then faced. That is, mentally trained because we had to think about a sequence of things’ (P17) |
| | Working on outcome performance indicators strengthens experiences with on the job training. | ‘I would ascribe it more on training, in the sense of the importance of training in and of itself. Bottom line, it is easy to write a protocol, but the difficulty is then to transmit it to the operators.’ (P18) |
| | Working on outcome performance indicators encourages a proactive approach to risk management. | ‘That is, if the good results with respect to certain clinical indicators are the result of a proactive approach to risk management and therefore of a culture based on the analysis of ... Elements that can lead to the occurrence of an event and get used to working in that way by the whole working group, in that sense there can be a positive influence…’ (P33) |
| | Good outcome performance indicators may imply more attention to infection prevention prior to Covid-19. | ‘…people who had already been trained and had in any case also had experience in managing other types of infections, isolations, who knew how to do a proper sanitation, both of the hands, even of the instruments, of the bed, of the patient living area, in fact this helped a lot.’ (P26) |
| | Good outcome performance indicators may imply less need for visits to emergency departments and hospitals, hence reducing Covid-19 exposure risk. | ‘certainly the good management prior to Covid, of pressure injuries, of infections […] probably allowed, we also thought, to limit access to the hospital, the emergency room.’ (P11) |
| *Possible explanation for the absence of an observable relationship* | | |
| | Covid-19 was a new challenge for which work on outcome performance indicators did not prepare. | ‘I completely disagree because they are two completely different aspects. Two events, two independent capacities.’ (P34) |
| **Availability of a quality officer** | | |
| *Possible explanation for the existence of an observable relationship* | | |
| | Often quality officers translate national and regional Covid-19 related guidelines and requirements into facility level protocols and procedures. | ‘Ok, regulations, ordinances were coming in, there was also the whole part relating to what was indicated by the Region to follow, etc. in the confusion of daily management. Having a quality manager who at that time was absolutely available |

| | |
|---|---|
| | also served us a bit as…' (P11) '…a guiding reference point.' (P112) |
| Quality officers might have a different perspective than general management or administrative staff when establishing new protocols and procedures. | 'then maybe the managers, the director ... are a little more sensitive to the bureaucratic aspect, and the relational aspect with the guest, with the relative or even with the care workers is lost a bit. Instead, the quality coordination figure, but also the nurse coordinator and care workers .... Well, in my opinion they are essential. I couldn't do without them.' (P20) |
| *Possible explanation for the absence of an observable relationship* | |
| If there is no quality officer, somebody else would perform the role of translating Covid-19 related guidelines and requirements. | 'There are two of us, plus the president of the board of directors, taking care of all this part here. So there is a formal appointment, but in my opinion […] What I think works is working together.' (P19) |
| There is no relation, because once a case is recognized, it is already too late for any intervention to have a meaningful effect. | 'The problem, in my opinion, lies in the fact that when a massive infection starts, or rather when I begin to notice the first case, […] I have already had it in the facility for four days. This means that even in a community in a bubble I will have the peak that will tend to give the maximum number of cases, regardless of this factor of having or not having the quality manager.' (P25) |
| **Quality certification of the facility with ISO 9001 or UNI 10881** | |
| *Possible explanation for the existence of an observable relationship* | |
| Helpful to the extent that contingency plans were required and prepared because of certification requirements | 'We already have evacuation plans for fire, earthquake and other emergencies and we also do various evacuation tests during the year. Surely it is a training that can be useful from all points of view.' (P17) |
| *Possible explanation for the absence of an observable relationship* | |
| The requirements of any certification system did not foresee Covid-19, so there were no related requirements | 'Even though one had been certified, there were additional unrequired things one had to cope with...'(P112) |
| **Availability of administrative software** | |
| *Possible explanation for the existence of an observable relationship* | |
| If an electronic health record is part of an administrative software, than the record indeed helps. | '…as far as the Covid management is concerned, however, the electronic health record has given, let's say ... It has absolutely reduced the waste of time by the staff in data management. So, all the |

| | |
|---|---|
| | time we manage to save is time dedicated to the patient, and to manage the situation.' (P18) |
| Perhaps facilities that don't have an administrative software were less accurate in reporting data. | 'It would be nice to know how much data was lost in those who were not using it.' (P16) |
| Correlation between size and availability of software. | 'Perhaps it is more probable that those who have administrative software are a larger structure and therefore, in most cases have larger outbreaks' (P19) |
| *Possible explanation for the absence of an observable relationship* | |
| A software does not help in Covid-19 related activities. | 'We, who have the electronic medical record, it's not like... It is certainly more convenient than the paper and all, but in relation to COVID it seems to me that ... Surely it is a useful, comfortable, faster, more dynamic device. But that's all.' (P20) |
| **Job satisfaction ratings** | |
| *Possible explanation for the existence of an observable relationship* | |
| Good relationships with management and within work teams likely means easier and quicker uptake of new procedures. | 'In places with a workplace climate where the person works well, feels good, certainly everything is easier, goals are reached more easily, etc.' (P27) |
| *Possible explanation for the absence of an observable relationship* | |
| Dedication of staff was essential, but this is not the same as satisfaction. | '…finding ourselves in the unusual and strange situation due to Covid, we had a beautiful demonstration [of dedication]' (P13) |
| The pandemic created a sense of responsibility among staff independent of job satisfaction. | 'I believe that not only the satisfaction of staff had influence, but another factor has also come into play, that is the professionalism, that cannot be taken away from care workers or nurses' (P37) |
| The pandemic created a sense of unity in the face of adversity independent of job satisfaction. | 'But, as far as the motivation is concerned, the group working climate, it almost seemed that Covid somehow favored this feeling of being on the same team.' (P27) |
| High turnover of staff may indicate that 2019 data are not really relevant. | 'The multifaceted, multi-standard and multi-strange staff that we -and perhaps also other nursing homes- have is a little wandering and a little wavering. And those from the cooperatives, especially the temporary workers, come and go.' (P13) |
| **Healthcare workers per available bed** | |
| *Possible explanation for the existence of an observable relationship* | |
| More personnel allows more extensive precautionary measures. | 'We needed [the extensive personnel] for 'my son had a contact' at a time when no one was vaccinated' (P11) |

| | |
|---|---|
| The establishment of "staff bubble" often requires a lot of personnel | 'Now we are finding ourselves, we have Covid in the nursing home, but not in [all the] services. So having enough staff to separate all services means a lot.' (P14) |
| *Possible explanation for the absence of an observable relationship* | |
| Number of personnel does not capture the dedication of staff. | 'it is not so much the availability of staff but the availability [in the sense of care] of the staff that has made the difference, especially at the time.' (P13) |
| Staff turnover is a problem because of the need for training and integration in work processes. | '…we engaged with the staff right away, with active training, continuous, during the shift and therefore unfaltering. Only to then start from scratch with new workers after the public calls [for personnel that were drained from the existing employees]' (P30) |
| Unclear testing rules for temporary personnel. | '[nursing home personnel] underwent periodic screening with antigenic swabs every 15 days, from 19 October 2020, something like that. […] at the [local health authority level, that provides external nurses] this did not exist. It was done on a voluntary basis. So we could get nurses who in reality were not subjected to any screening.' (P24) |
| Once personnel is infected, you have many absences. | 'The difficult thing at that moment was also the liberation, for lack of a better term, of the person who becomes Covid negative. Because at the time it was different from now, when the end of isolation comes immediately, […] then there were biblical time intervals before the worker could work again.' (P26) |
| **Availability of an isolation area** | |
| *Possible explanation for the existence of an observable relationship* | |
| The isolation area was used for quarantine. | 'However, I repeat, due to the fact that new admission or patients returning from a hospitalization were put there [in the isolation area] in my opinion, in a certain sense, in a certain sense it helped' (P14) |
| *Possible explanation for the absence of an observable relationship* | |
| Having an isolation room is not sufficient to isolate residents, the facility's architecture is often a limiting factor. | 'One example is the management of the rooms' bathrooms. One of the biggest problems here was the room with the bathroom inside. So the rooms had a dedicated bathroom, but it wasn't ... Not all of them were directly accessible from inside the room' (P17) |
| Strict isolation is not really feasible in a nursing home, with or without an isolation room | 'We were organized to be permeable to the surrounding community. So there is no way to isolate. You can't become an intensive care unit overnight. We are not.' (P19) |
| Once a case is identified the virus has already spread in the facility | 'when we realize that there is a suspected case or when it is confirmed by a positive swab, at that |

| | |
|---|---|
| | point the spread of the virus has already occurred in the others and therefore it is not those two rooms [for isolation] that guarantee the possibility that the virus is contained' (P33) |

*Quotes translated by the authors
